# Anthocyanins increase serum adiponectin in newly diagnosed diabetes but not in prediabetes: a randomized controlled trial

**DOI:** 10.1186/s12986-020-00498-0

**Published:** 2020-09-21

**Authors:** Liping Yang, Wenhua Ling, Yun Qiu, Yong Liu, Li Wang, Jing Yang, Changyi Wang, Jianping Ma

**Affiliations:** 1 Center for Chronic Disease Control, NanShan, Shenzhen, People’s Republic of China; 2 Guangdong Provincial Key Laboratory of Food, Nutrition and Health, Department of Nutrition, School of Public Health, Sun Yat-Sen University, Guangzhou, People’s Republic of China

**Keywords:** Anthocyanin, Adiponectin, Prediabetes

## Abstract

**Background:**

Epidemiological studies have suggested that adiponectin is associated with the development of insulin resistance and type 2 diabetes. This study first examined the effect of purified anthocyanins, a group of dietary flavonoids, on serum adiponectin in patients with prediabetes and newly diagnosed diabetes.

**Methods:**

A total of 160 patients with prediabetes (n = 90) or newly diagnosed diabetes (n = 70) were randomly assigned to either the anthocyanins group or the placebo group for 12 weeks of intervention. Serum adiponectin, a set of biomarkers related to glucolipid metabolism, anthropometric parameters, dietary intake and physical activity were measured before and after intervention.

**Results:**

Anthocyanins increased serum adiponectin compared with placebo (net change 0.46 µg/mL, 95% CI [0.03, 0.90], *p* = 0.038) in the subjects with newly diagnosed diabetes. No significant difference in the change in adiponectin was observed between the two groups either in the overall subjects (0.02 µg/mL [− 0.32, 0.36], *p* = 0.906) or in prediabetes (− 0.35 µg/mL [− 0.85, 0.16], *p* = 0.174). Anthocyanins also decreased fasting glucose (− 0.5 mmol/L [− 1, − 0.04], *p* = 0.035) in the subjects with newly diagnosed diabetes, but no such change was observed in those with prediabetes.

**Conclusions:**

Anthocyanins supplementation for 12 weeks improved serum adiponectin and fasting glucose in patients with newly diagnosed diabetes, but not in patients with prediabetes.

**Trial registration:**

ClinicalTrials.gov, NCT02689765. Registered on 6 February 2016, https://clinicaltrials.gov/ct2/show/NCT02689765.

## Highlights

- The First study to examine the effects of purified anthocyanins on serum adiponectin in patients with prediabetes and newly diagnosed diabetes.
- Anthocyanins increased serum adiponectin and decreased fasting glucose in patients with newly diagnosed diabetes, but not in prediabetes.
- Provide new evidence for anthocyanins in the treatment and prevention of type 2 diabetes.

## Introduction

The prevalence of type 2 diabetes mellitus (T2DM) in conjunction with its cardiovascular complications is becoming the most serious health challenge worldwide. Adiponectin, the most common adipokine, has been suggested to exert antiatherogenic and anti-inflammatory activities in various cardiometabolic diseases. It is also known to be an important regulator of insulin sensitivity and glucose metabolism. A previous meta-analysis conclusively showed a strong and consistent correlation between higher circulating adiponectin levels and lower risk of T2DM [1]. Another recent meta-analysis showed that hypoadiponectinemia was associated with the development of T2DM [2]. Adiponectin has been proposed to be a therapeutic target and prognostic marker for cardiometabolic diseases [3]. However, there are many conflicting results about the associations between circulating adiponectin and the prognosis of T2DM [4–7]. To date, the complex interplay between adiponectin and T2DM is not fully understood, and the role of circulating adiponectin in the development of diabetes remains unclear.

Anthocyanins are a large group of phytochemicals, concentrated from dark fruits and vegetables and are associated with health benefits. A cross-sectional study suggested that higher anthocyanin intake was associated with lower peripheral insulin resistance, reduced levels of inflammatory markers and improved adiponectin concentrations [8]. Numerous interventional studies have revealed that anthocyanins or anthocyanin-rich extracts improve lipid profiles in cardiometabolic diseases, but few studies have evaluated the effect of anthocyanins on circulating adiponectin [9, 10]. Only one study examined the effect of anthocyanins on serum adiponectin in patients with well-controlled type 2 diabetes, and anthocyanins were found to increase adiponectin and decrease fasting glucose [11]. Whether these effects could be extended to patients with prediabetes or untreated newly diabetes is unclear. Prediabetes or newly diagnosed diabetes are identified to avoid the influence of medications and are more appropriate to prospectively evaluate the efficacy of anthocyanins intervention. Therefore, the aim of this study was to evaluate the effect of purified anthocyanins supplementation on serum adiponectin levels in patients with prediabetes or newly diagnosed diabetes.

## Methods

### Subjects

Participants aged 40–75 years were recruited from local community at outpatient clinic of a hospital in Guangzhou city, China. Potential participants were initially screened by their recent fasting blood glucose records, and volunteers were further screened by a face-to-face interview and were given a 3-h 75 g oral glucose tolerance test (OGTT) to confirm their eligibility. Patients with prediabetes or newly diagnosed diabetes were eligible for this study. According to the diagnostic criteria of the American Diabetes Association (ADA) [12], prediabetes is an intermediate state in which subjects meet one of the following criteria: impaired fasting glucose (IFG, 5.6–6.9 mmol/L), impaired glucose tolerance (IGT, 2-h glucose 7.8–11.0 mmol/L), or a glycated hemoglobin A1c (HbA1c) level of 5.7–6.4%. Newly diagnosed diabetes was diagnosed in subjects who exceeded the upper limit of the above criteria (fasting glucose > 6.9 mmol/L, 2-h glucose > 11.0 mmol/L, or HbA1c > 6.4%), and had not taken any diabetic medications before the screening.

Participants were excluded if they had pre-existing diabetes, had a history of hypoglycemic medical treatment, acute or chronic infectious diseases, untreated thyroid disease, serious liver or kidney dysfunction, the use of glucocorticoids, or suffering from traumatic injury or undergoing surgery within 6 months before enrollment; were lactating or pregnant women; or were individuals with polycystic ovarian syndrome. This trial was approved by the Ethics Committee of Sun Yat-Sen University, and written informed consent was obtained from each participant before enrollment.

### Study design

This study was a 12-week randomized placebo-controlled trial that was registered at ClinicalTrials.gov as NCT02689765. The primary outcome was change in serum adiponectin. Sample size calculation were based on the previous study using purified anthocyanins as supplements [11], in which the change in serum adiponectin was 1.2 µg/mL (SD 1 µg/mL). We calculated that 60 participants (prediabetes or newly diagnosed diabetes) per group, would be enough to detect a significant change in adiponectin (1 µg/mL, SD 2 µg/mL) at 80% power and an alpha level of 0.05. Considering a 20% dropout rate, we planned to recruit 80 participants per group and a total of 160 participants in this study.

Eligible participants were randomly assigned to either the anthocyanins group (n = 80) or the placebo group (n = 80). The allocation sequences were determined by a computer-generated random-numbers table. The anthocyanins group consumed two anthocyanin capsules (Medox; Polyphenols AS, Norway) twice daily for a total of 320 mg anthocyanins. The dose of anthocyanins was determined based on our previous trials that were performed in patients with T2DM [11] and dyslipidemia [13]. The control group consumed two identically packaged placebo capsules twice daily.

The participants were asked to maintain their habitual diet and physical activities, and to avoid consuming anthocyanin-rich foods, such as berries and grapes, during the whole study. Dietary analyses were conducted at baseline and 12 weeks of the study. Subjects were asked to provide detailed 3-day food records, and they were guided by a trained investigator. Scheduled capsules were dispensed to the participants, and participants were asked to return any remaining capsules to the clinic every 2 weeks. Compliance was assessed by using a short questionnaire and counting their remaining capsules. When the subjects consumed fewer than 80% of the dispensed capsules, they were excluded from the trial.

### Outcome measures

At baseline and at the end of intervention, height and weight were measured by trained staff, and body mass index (BMI) was calculated as weight divided by height squared (kg/m^2^). Waist circumference, hip circumference and blood pressure were measured by the same staff. Fasting blood samples were collected after fasting overnight, and a 3-h OGTT was performed (blood samples were collected at 30, 60, 120, and 180 min after a 75 g glucose challenge).

Serum samples were centrifuged (15 min, 3000×*g*, 4 °C) within 30 min after blood collection and were stored at − 80 °C. Fasting serum adiponectin was measured using an ELISA kit with a solid phase two-site enzyme immunoassay (Mercodia, Uppsala, Sweden). The average intra- and inter-assay coefficients of variation for adiponectin were 3.1 and 5.4%, respectively.

HbA1c was analyzed by high-pressure liquid chromatography (HPLC) (Bio-Rad Laboratories, USA). Insulin and C-peptide were assayed by electrochemical luminescence (Roche Diagnostics, Indianapolis, USA). Laboratory analyses of blood glucose, lipid profiles (total cholesterol, triglycerides, high-density lipoprotein cholesterol, low-density lipoprotein cholesterol, apolipoprotein A-1 and apolipoprotein B), C-reactive protein (CRP), and safety variables (liver enzymes) were performed by using an automatic biochemical analyzer (Mindray BS600, Shenzhen, China) following standard protocols.

### Statistical analysis

SPSS 22.0 (IBM Inc., Chicago, IL, USA) was used for statistical analyses, and R 3.5.3 (R Foundation, Vienna, Austria) was used for graphing. *P* < 0.05 was considered significant. The per-protocol data set without the imputation of missing data was used for data analysis. We used the unpaired Student’s *t*-test (for continuous variables) and Chi-squared test (for categorical variables) to evaluate the baseline differences between the anthocyanins and the placebo groups. An independent Student’s *t*-test was used to compare the differences between the two groups after intervention. We assessed the differences before and after the intervention within each group by using a paired *t*-test. Multiple comparisons were statistically corrected by using the false discovery rate.

## Results

### General characteristics of the subjects

**Fig. 1:**
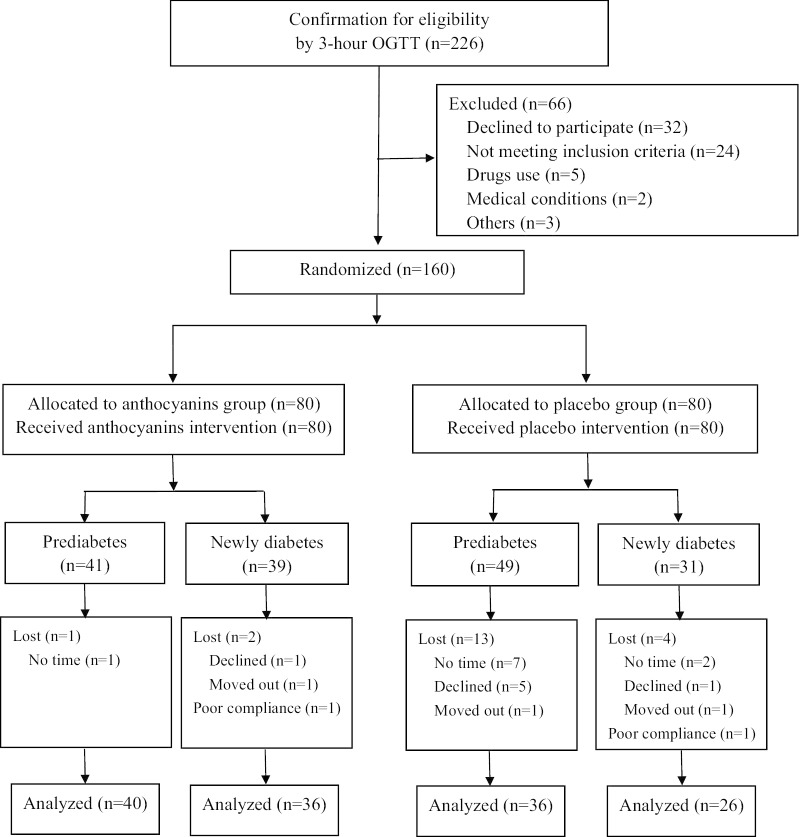
The CONSORT flow diagram for participants

A total of 160 eligible participants (80 in each intervention group) with prediabetes (n = 90) or newly diagnosed diabetes (n = 70) were included in this trial. The baseline characteristics of all the randomized participants are presented in Additional file 1: Table S1. Participants in the anthocyanins and placebo groups were comparable in terms of age, gender, education, lifestyle factors, dietary intake and physical activity. Twenty participants (12.5%) were lost from the study and two participants (one each group) were in poor compliance, resulting in 138 patients finally analyzed. More dropouts were observed in the placebo group (n = 17) than in the anthocyanins group (n = 3), with response rates of 79% and 96%, respectively. The flow chart presented in Fig. 1 shows the allocation and the numbers of patients ultimately available for each group.

More than 90% of the participants in each group consumed at least 85% of the supplements provided, and no significant difference was observed in the proportion of poor compliance between the two groups. Participants reported a total of 10 adverse events to the intervention, and the proportion of adverse events was not significantly different between the two groups (Additional file 1: Table S2). Three adverse events were reported among participants in the placebo group, including abdominal pain (n = 1), diarrhea (n = 1) and skin rash (n = 1); seven adverse events were reported among participants in the anthocyanins group, including black stool (n = 5), insomnia (n = 1) and dizziness (n = 1). Within the subgroup of prediabetes and newly diagnosed diabetes, comparisons of baseline values indicated that the subjects in the two intervention groups had similar adiponectin levels and metabolic parameters (Table 1). No significant difference was observed between the anthocyanins and the placebo groups in terms of dietary intake and nutrients at either baseline or study end (Additional file 1: Table S3).

**Table 1:** Baseline values of adiponectin and glucolipid metabolic parameters in the anthocyanins and placebo group for the participants classified with prediabetes and newly diabetes

	Prediabetes			Newly diabetes		
	Anthocyanins (n = 41)	Placebo (n = 49)	*P*	Anthocyanins (n = 39)	Placebo (n = 31)	*P*
Adiponectin (µg/mL)	6.05 ± 2.48	6.49 ± 2.28	0.41	5.93 ± 2.4	5.77 ± 1.62	0.762
*Glucose metabolism*						
Hemoglobin A1c (%)	5.8 ± 0.42	5.63 ± 0.43	0.074	6.49 ± 0.53	6.46 ± 0.48	0.854
Fasting glucose (mmol/L)	5.68 ± 0.68	5.87 ± 0.44	0.145	6.62 ± 0.82	6.48 ± 0.66	0.418
2-h glucose (mmol/L)	7.66 ± 2.03	7.05 ± 1.8	0.144	12.08 ± 3.03	11.74 ± 3.34	0.661
Fasting insulin (μU/mL)	11.58 ± 5.79	11.22 ± 5.6	0.773	11.49 ± 7.12	13.21 ± 6.6	0.314
2-h insulin (μU/mL)	81.45 ± 53.51	82.12 ± 71.98	0.961	102.56 ± 68.11	109.42 ± 71.86	0.686
Fasting C-peptide (ng/mL)	2.27 ± 0.92	2.29 ± 0.81	0.943	2.58 ± 1.1	2.81 ± 1.26	0.424
2-h C-peptide (ng/mL)	10.9 ± 3.68	10.56 ± 3.7	0.674	12.14 ± 3.86	12.45 ± 4.25	0.754
AUC glucose	23.7 ± 4.04	23.96 ± 3.76	0.785	33.66 ± 6.01	32.56 ± 5.55	0.465
AUC insulin	195.77 ± 95.64	208.35 ± 105.93	0.608	211.23 ± 126.83	260.78 ± 129.8	0.142
AUC C-peptide	25.72 ± 7.44	25.47 ± 6.22	0.875	26.64 ± 7.35	29.86 ± 8.47	0.121
HOMA-IR	2.94 ± 1.6	2.91 ± 1.39	0.937	3.41 ± 2.39	3.91 ± 2.18	0.383
HOMA-β	112.65 ± 68.82	104.3 ± 97.25	0.664	77.31 ± 41.61	87.36 ± 38.99	0.316
*Lipids*						
Total cholesterol (mmol/L)	5.99 ± 1.2	5.99 ± 1.03	0.996	6.24 ± 1.17	6.06 ± 1.46	0.57
Triglycerides (mmol/L)	1.7 ± 0.9	1.69 ± 1	0.949	1.73 ± 1.14	1.8 ± 1.58	0.822
HDL cholesterol (mmol/L)	1.45 ± 0.39	1.48 ± 0.34	0.693	1.48 ± 0.37	1.43 ± 0.34	0.528
LDL cholesterol (mmol/L)	3.35 ± 0.88	3.28 ± 0.82	0.67	3.43 ± 0.94	3.33 ± 0.99	0.654
Apo A-1 (g/L)	1.55 ± 0.3	1.66 ± 0.28	0.073	1.64 ± 0.34	1.63 ± 0.34	0.907
Apo B (g/L)	1.17 ± 0.25	1.13 ± 0.22	0.506	1.16 ± 0.22	1.14 ± 0.28	0.836
LDLHDL ratio	2.44 ± 0.76	2.34 ± 0.82	0.554	2.46 ± 0.86	2.46 ± 0.94	0.998
TGHDL ratio	1.33 ± 0.93	1.29 ± 1.08	0.848	1.32 ± 1.21	1.42 ± 1.49	0.77

AUC, area under the curve by 3-h oral glucose tolerance test, were calculated according the trapezoidal rule

HOMA-IR, homoeostasis model assessment of insulin resistance; HOMA-IR = FIns (mU/mL) × FG (mmol/L)/22.5

HOMA-β, homoeostasis model assessment of β-cell function; HOMA-β = FIns × 20/(FG-3.5)

*HDL* high-density lipoprotein, *LDL* low-density lipoprotein, *apo A-1* apolipoprotein A-1, *apo B* apolipoprotein B, *TG* Triglycerides

*P* value was for the differences between the two groups, compared by independent student’s *t*-test

### Changes in serum adiponectin

**Fig. 2:**
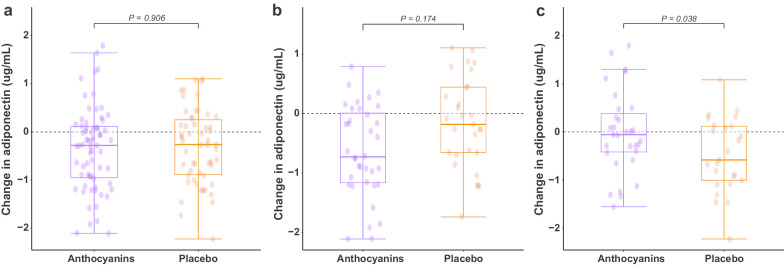
Changes in serum adiponectin after 12 weeks of intervention with anthocyanins or placebo. **a** changes in serum adiponectin among all subjects, **b** changes in serum adiponectin in the subjects with prediabetes, **c** changes in serum adiponectin in the subjects with newly diagnosed diabetes

After 12 weeks of intervention, serum adiponectin was reduced in both the placebo group (− 0.31 µg/mL [− 0.55, − 0.06], *p* = 0.015) and the anthocyanins group (− 0.29 µg/mL [− 0.53, − 0.05], *p* = 0.021). No significant difference in the net change in serum adiponectin was observed between the anthocyanins and the placebo group among all subjects (0.02 µg/mL [− 0.32, 0.36], *p* = 0.906) (Fig. 2a).

In the subgroup with prediabetes, serum adiponectin was reduced in the anthocyanins group (− 0.51 µg/mL [− 0.86, − 0.16], *p* = 0.006) but not in the placebo group (− 0.17 µg/mL [− 0.53, 0.2], *p* = 0.365), and the net change between the two groups was nonsignificant (− 0.35 µg/mL [− 0.85, 0.16], *p* = 0.174) (Fig. 2b). In the subgroup with newly diagnosed diabetes, there was a significant reduction in serum adiponectin in the placebo group (− 0.48 µg/mL [− 0.8, − 0.16], *p* = 0.004), whereas there was no change in the anthocyanins group (− 0.02 µg/mL [− 0.33, 0.3], *p* = 0.917), and the net change in adiponectin between the two groups was statistically significant (0.46 µg/mL [0.03, 0.90], *p* = 0.038) (Fig. 2c).

### Changes in markers for glucose and lipid metabolism

Compared with placebo, anthocyanins decreased fasting glucose (− 0.5 mmol/L [− 1, − 0.04], *p* = 0.035) in the subjects with newly diagnosed diabetes (Additional file 1: Table S4), increased serum apolipoprotein A-1 (apo A-1) **(**0.15 g/L [0.03, 0.26], *p* = 0.016) and decreased apolipoprotein B (apo B) (− 0.1 g/L [− 0.17, − 0.03], *p* = 0.008) in the subjects with prediabetes (Additional file 1: Table S5). Similar changes in apo A-1 and apo B were observed among all subjects. No significant change was observed in inflammatory markers (such as C-reactive protein), the anthropometric measurements (such as body weight, BMI, waist and hip circumferences), or blood pressure either among all subjects or among the subgroups (data available upon request).

## Discussion

To our knowledge, this is the first study to examine the effect of supplementation with purified anthocyanins on serum adiponectin in patients with prediabetes and newly diagnosed diabetes. This study showed that supplementation with 320 mg/day of purified anthocyanins for 12 weeks improved serum adiponectin and decreased fasting glucose, independent of hypoglycemic agents, in patients with newly diagnosed diabetes but not in patients with prediabetes.

### Comparison with other studies

Few intervention studies have investigated the effect of anthocyanins on circulating adiponectin in cardiometabolic diseases. A previous trial from our laboratory conducted in patients with well-controlled type 2 diabetes showed that purified anthocyanins increased serum adiponectin and decreased fasting glucose, but potential interactions with hypoglycemic medications were not avoided [11]. Thus, we rigorously tried to ensure that our participants did not take any hypoglycemic drug or present with any other chronic inflammatory disease that could interfere with the results. In the present study, we observed significant improvements in adiponectin and fasting glucose in the participants with newly diagnosed diabetes but not in those with prediabetes, which may be due to the lower hyperglycemic status of prediabetes.

Three trials reported changes in adiponectin using anthocyanin-rich supplements in patients with metabolic syndrome. Black raspberry treatment for 12 weeks significantly improved serum adiponectin levels (net change 1.9 µg/mL) and vascular endothelial function [10]. Consuming cranberry juice for 60 days increased serum adiponectin levels (graphic display) but had no effect on inflammatory markers [9]. Anthocyanin-rich grape powder increased plasma adiponectin in subjects with nondyslipidemia (net change 1.1 µg/mL) but decreased plasma adiponectin in subjects with dyslipidemia (net change − 1.7 µg/mL), who might have increased levels of inflammation and be less responsive to the dietary intervention [14]. Our participants had a mean triglyceride level of 1.8 mmol/L and a mean HDL level of 1.46 mmol/L, with approximately 50% being within the metabolically normal range; moreover, they had a normal mean CRP level of 2.2 mg/L. It is not surprising to observe a less remarkable improvement in adiponectin (net change 0.46 µg/mL) in the subjects with newly diagnosed diabetes and a nonsignificant change in prediabetes and among all subjects.

### Possible mechanism

It has been demonstrated that adiponectin could increase hepatic insulin sensitivity and glucose utilization through the activation of adenosine monophosphate (AMP) kinase and peroxisome proliferator-activated receptor-alpha (PPARα) [15]. A previous experimental study from our laboratory showed that anthocyanin upregulated adiponectin secretion in adipocytes through the transcription factor forkhead box O1 (FoxO1) in diabetic mice [16]. Basic studies have also demonstrated the antidiabetic effect of anthocyanins by activating AMP-activated protein kinase and PPAR in adipose tissue and skeletal muscles [17]. Anthocyanin might also ameliorate insulin resistance via the activation of insulin signaling and enhanced glucose transporter 4 (GLUT4) translocation, which increases glucose uptake and reduces the hyperglycemia associated with metabolic disorders [18]. These studies may help to explain the alterations of adiponectin and fasting glucose in patients with newly diagnosed diabetes.

The potential cardiovascular-protective effect of anthocyanins is clearly understood. The study of our laboratory also found that anthocyanin protected against diabetes-related endothelial dysfunction [16]. On the other hand, hypoadiponectinemia-induced inflammasome activation may be the molecular mechanism for diabetic vascular endothelial dysfunction [19]. Basic and clinical studies have identified the anti-atherogenic function of adiponectin [20] and its positive effect on vascular inflammation and endothelial function [21]. Adiponectin could also improve endothelium-independent vasodilation by inducing the production of endothelial nitric oxide (NO) [22].

### Clinical implications of the findings

In the patients with newly diagnosed diabetes, we observed a decrease in serum adiponectin in the placebo group, which revealed a change in adiponectin in the progression of T2DM, whereas anthocyanins prevented the decline in adiponectin, leading to a mild but significant relative incremental improvement. Epidemiological studies have suggested that hypoadiponectinemia is an important component of the pathogenesis in insulin resistance and T2DM [23]. A cross-sectional study suggested that decreased serum adiponectin might be an independent predictor for the progression of T2DM [24]. Some prospective studies have shown that low levels of adiponectin predict the development of insulin resistance [25, 26]. Adiponectin has been proposed to be a novel target for the prevention and treatment of T2DM [27, 28]. Taken together, anthocyanins may prevent the progression of T2DM by improving serum adiponectin and fasting glucose in newly diagnosed diabetes.

Several studies suggested that hypoadiponectinemia was associated with vascular damage and contributed to cardiovascular risk in T2DM [29, 30]. A cohort study showed that a low level of adiponectin might predict the impairment of endothelium-independent vasodilation in newly diagnosed T2DM patients [31]. Thus, the favorable improvement in adiponectin in this study may also have a cardiovascular-protective effect, which is consistent with the clinical implications of the improvements in apo A-1 and apo B [32, 33].

### Limitations

The following limitations should be considered in the present study: first, there was possible incomplete blinding in this study because of the different contents of capsules, which may have resulted in the relatively high dropout in the placebo group; second, there was no objective measure of compliance during the intervention period, and data on serum anthocyanins or their metabolites were lacking. The time required for complete clearance of anthocyanins from the circulation is 6–8 h, which is exceeded by the 8–10 h of fasting [34]. Finally, we only measured total adiponectin rather than high-molecular-weight adiponectin. Nonetheless, data on the relevance of their distinction showed similar results [35, 36].

In conclusion, this randomized placebo-controlled trial showed that 12 weeks of daily supplementation with 320 mg anthocyanins increased serum adiponectin and decreased fasting glucose in patients with newly diagnosed diabetes but not in those with prediabetes. Additional long-term studies are needed to confirm the serial changes in adiponectin by anthocyanins intervention from normoglycemic individuals to individuals with prediabetes and new-onset diabetes.

## Supplementary information

**Additional file 1**: **Table S1**: Baseline characteristic of the participants; **Table S2**: The adverse events reported in the trial; **Table S3**: Average daily intake of food groups and nutrients by the participants at baseline and 12 weeks; **Table S4**: Changes in adiponectin and glucolipid metabolic markers after 12-week intervention in subjects with newly diagnosed diabetes; **Table S5**: Changes in adiponectin and glucolipid metabolic markers after 12-week intervention in subjects with prediabetes.

## Data Availability

The datasets used and/or analyzed during the current study are available from the corresponding author on reasonable request.

## Abbreviations

T2DM: Type 2 diabetes
RCT: Randomized controlled trial
IFG: Impaired fasting glucose
IGT: Impaired glucose tolerance
HbA1c: Glycated hemoglobin A1c
LDL: Low-density lipoprotein
HDL: High-density lipoprotein
CRP: C-reactive protein
apoA-1: Apolipoprotein A-1
apo B: Apolipoprotein B
