# Supplementary material for: Anthocyanins increase serum adiponectin in newly diagnosed diabetes but not in prediabetes: a randomized controlled trial
Source: Nutr Metab (Lond). 2020 Sep 21;17:78. doi: 10.1186/s12986-020-00498-0 (PMC7507266; doi:10.1186/s12986-020-00498-0)
Supplement: Supplementary file 1 — Additional file 1: Table S1: Baseline characteristic of the participants; Table S2: The adverse events reported in the trial; Table S3: Average daily intake of food groups and nutrients by the participants at baseline and 12 weeks; Table S4: Changes in adiponectin and glucolipid metabolic markers after 12-week intervention in subjects with newly diagnosed diabetes; Table S5: Changes in adiponectin and glucolipid metabolic markers after 12-week intervention in subjects with prediabetes. [file 12986_2020_498_MOESM1_ESM.docx]

**Table S1: Baseline characteristic of the participants**

|  | **Anthocyanins(n=80)** | **Placebo(n=80)** | ***P*** |
| --- | --- | --- | --- |
| **Demographics** |  |  |  |
| Age (years) | 60.8±7.9 | 61.2±6.9 | 0.724 |
| Gender (Male/Female） | 25/55 | 29/51 | 0.504 |
| Education |  |  | 0.676 |
| Primary school | 5(6.3%) | 8(10%) |  |
| Middle school | 51(63.7%) | 48(60%) |  |
| College | 24(30%) | 24(30%) |  |
| Occupation |  |  | 0.207 |
| Professional laborers | 41(51.3%) | 43(53.8%) |  |
| Physical laborers | 33(41.2%) | 25(31.2%) |  |
| Others | 6(7.5%) | 12(15%) |  |
| **Lifestyles** |  |  |  |
| Current Smoking | 12(15%) | 8(10%) | 0.339 |
| Regular alcohol drinking | 4(5%) | 4(5%) | 1.000 |
| Sports |  |  | 0.516 |
| 1~3 times/week | 29(36.3%) | 33(42.3%) |  |
| 4~7times/week | 51(63.7%) | 47(58.7%) |  |
| **Anthropometrics** |  |  |  |
| Weight (Kg) | 63.7±11.9 | 63.1±10. 5 | 0.742 |
| BMI (Kg/m^2^) | 24.7±3.2 | 24.8±3.4 | 0.762 |
| Waist circumference (cm) | 87.8±9.3 | 88.0±8.9 | 0.880 |
| Waist to hip ratio | 0.905±0.054 | 0.905±0.055 | 0.981 |
| **Blood pressures** |  |  |  |
| SBP (mmHg) | 133.1±16.5 | 133.0±21.2 | 0.977 |
| DBP (mmHg) | 78. 8±9. 9 | 78.7±9.5 | 0.950 |

Data are presented as mean ±standard deviation for continuous variables and n (%) for categorical variables. *P* value was for the differences between the two groups, compared by independent Student’s t-test or chi-square test. BMI, body mass index; SBP: systolic blood pressure. DBP diastolic blood pressure.

**Table S2: The adverse events reported in the trial**

| **Adverse events** | **Anthocyanins** | **Placebo** |
| --- | --- | --- |
| Abdominal pain | 0 | 1 |
| Diarrhea | 0 | 1 |
| Skin rash | 0 | 1 |
| Dizziness | 1 | 0 |
| Insomnia | 1 | 0 |
| Black stool | 5 | 0 |
| **Total** | 7 | 3 |

**Table S3**: **Average daily intake of food groups and nutrients by the participants at baseline and 12 weeks**

|  | **Anthocyanin (n=76)** | | **Placebo (n=62)** | | ***P*** |
| --- | --- | --- | --- | --- | --- |
|  | **Baseline** | **12 weeks** | **Baseline** | **12 weeks** |  |
| **Food groups (g/d)** | | | | | |
| Grain, Cereal Bread | 372.31±214.71 | 384.67±200.20 | 386.14±161.45 | 352.21±151.28 | 0.201 |
| Poultry, Meat | 99.56±66.67 | 110.82±55.36 | 113.9±81.12 | 100.39±46.54 | 0.072 |
| Fish, Shrimps | 68.96±67.57 | 56.18±57.40 | 67.9±67.35 | 65.89±57.16 | 0.419 |
| Eggs | 18.72±24.78 | 23.24±28.58 | 17.78±19.49 | 28.93±28.22 | 0.202 |
| Milk products | 70.44±106.43 | 75.67±96.42 | 62.53±92.05 | 75.98±121.83 | 0.670 |
| Soy products | 5.49±13.29 | 10.15±24.05 | 17.39±29.53 | 18.84±54.72 | 0.666 |
| Vegetables | 318.46±206.31 | 297.66±189.64 | 336.71±203.51 | 308.44±169.48 | 0.832 |
| Fruits | 88.45±180.47 | 91.18±87.13 | 90.99±124.29 | 95.56±117.20 | 0.948 |
| **Nutrients** | | | | | |
| Energy (kcal/d) | 1753.46±707.57 | 1764.53±595.63 | 1794.95±702.24 | 1754.21±643.61 | 0.704 |
| Protein (g/d) | 106.93±46.93 | 110.95±44.78 | 113.43±51.96 | 111.86±49.98 | 0.590 |
| Carbohydrate (g/d) | 271.02±137.12 | 277.95±120.8 | 279.06±109.2 | 262.54±99.78 | 0.312 |
| Fat (g/d) | 51.79±29.36 | 54.61±34.01 | 50.74±27.16 | 58.79±39.13 | 0.466 |
| Cholesterol (mg/d) | 271.02±137.12 | 277.95±120.8 | 279.06±109.2 | 262.54±99.78 | 0.477 |
| Vitamin A (IU/d) | 502.96±342.22 | 493.91±252.29 | 522.49±277.46 | 521.94±238.33 | 0.874 |
| Vitamin E (mg/d) | 20.35±26.59 | 19.97±13.34 | 21±19.77 | 22.32±19.1 | 0.695 |
| Vitamin C (mg/d) | 59.98±39.75 | 56.31±34.22 | 63.34±37.41 | 58.39±31.19 | 0.843 |
| Fiber (g/d) | 16.22±11.16 | 15.73±6.22 | 16.94±8.86 | 16.65±8.02 | 0.907 |
| Anthocyanins (mg/d) | 10.06±6.8 | 9.91±3.67 | 10.53±5.12 | 9.99±4.38 | 0.697 |

Data are analyzed using the per-protocol data set and are presented as mean ±standard deviation. Food groups and nutrients were calculated based on 3-day dietary records at baseline and at 12 weeks. The anthocyanins only represent the subjects’ daily dietary intake, it does not include the supplemental anthocyanin dose provided.

*P* value was for the comparisons of changes between the anthocyanins and placebo groups by independent Student’s *t*-test.

**Table S4: Changes in adiponectin and glucolipid metabolic markers after 12-week intervention in subjects with newly diagnosed diabetes**

|  | **Anthocyanins(n=36)** | | **Placebo(n=26)** | | **Net change**  **(95% CI)** | ***P t-test*** |
| --- | --- | --- | --- | --- | --- | --- |
|  | **Baseline** | **12 weeks** | **Baseline** | **12 weeks** |  |  |
| Adiponectin (ug/mL) | 5.72±1.63 | 5.71±1.63 | 5.79±1.7 | 5.31±1.5 | 0.46(0.03, 09) ^b^ | 0.038 |
| **Glucose metabolism** |  |  |  |  |  |  |
| Hemoglobin A1c (%) | 6.52±0.55 | 6.15±0.41 ^a^ | 6.45±0.52 | 6.2±0.55 ^a^ | -0.12 (-0.32, 0.07) | 0.214 |
| Fasting glucose (mmol/L) | 6.68±0.8 | 6.42±0.71 | 6.51±0.7 | 6.75±0.68 | -0.5 (-1, -0.04) ^b^ | 0.035 |
| 2-h glucose (mmol/L) | 12.29±3.12 | 12.07±2.99 | 12.2±3.45 | 12.41±3.04 | -0.43 (-2.21, 1.35) | 0.63 |
| Fasting insulin (μU/mL) | 11.86±7.26 | 11.16±5.9 | 13.23±6.53 | 13.98±8.26 | -1.92 (-4.64, 0.8) | 0.163 |
| 2-h insulin (μU/mL) | 106.31±70.89 | 108.64±55.3 | 100.53±68.02 | 126.88±96.78 ^a^ | -20.76 (-52, 10.47) | 0.188 |
| Fasting C-peptide (ng/mL) | 2.65±1.11 | 2.57±0.89 | 2.84±1.21 | 2.93±1.09 | -0.21 (-0.61, 0.18) | 0.284 |
| 2-h C-peptide (ng/mL) | 12.41±3.98 | 12.58±2.89 | 12.34±4.02 | 13.66±4.33 ^a^ | -0.84 (-2.23, 0.56) | 0.235 |
| AUC Glucose | 33.94±6.32 | 33.56±6.18 | 33.44±5.59 | 34.73±4.59 | -1.52 (-5.08, 2.03) | 0.392 |
| AUC Insulin | 218.37±135.51 | 221.21±96.36 | 230.94±121.58 | 278.82±161.57 ^a^ | -41.73 (-97.84, 14.38) | 0.141 |
| AUC C-peptide | 27.06±7.89 | 27.1±5.35 | 28.18±8.58 | 30.65±8.73 ^a^ | -2.03 (-4.83, 0.78) | 0.153 |
| HOMA-IR | 3.56±2.45 | 3.2±1.72 | 3.99±2.26 | 4.28±2.66 | -0.75 (-1.75, 0.25) | 0.14 |
| HOMA-β | 77.72±41.6 | 80.54±45.13 | 84.46±33.86 | 85.1±47.29 | 2.16 (-15.99, 20.32) | 0.812 |
| **Lipids** |  |  |  |  |  |  |
| Total cholesterol (mmol/L) | 6.21±1.24 | 6.02±1.26 | 6.14±1.53 | 6.44±1.7 | -0.47(-1.16,0.22) | 0.176 |
| Triglycerides (mmol/L) | 1.77±1.2 | 1.79±0.96 | 1.92±1.74 | 2.52±2.34 | -0.59(-1.53,0.35) | 0.211 |
| HDL cholesterol (mmol/L) | 1.48±0.37 | 1.28±0.36 ^a^ | 1.45±0.35 | 1.26±0.37 ^a^ | -0.03(-0.15,0.1) | 0.677 |
| LDL cholesterol (mmol/L) | 3.39±0.95 | 3.09±0.64 | 3.35±1.07 | 3.26±1.02 | -0.23(-0.64,0.18) | 0.285 |
| Apo A-1 (g/L) | 1.65±0.33 | 1.62±0.3 | 1.68±0.36 | 1.55±0.27 | 0.12(-0.04,0.28) | 0.134 |
| Apo B (g/L) | 1.14±0.2 | 1.1±0.19 | 1.13±0.27 | 1.15±0.37 | -0.06(-0.19,0.08) | 0.39 |

Data are analyzed using the per-protocol data set and are presented as mean ±standard deviation.

AUC, area under the curve by 3-hour oral glucose tolerance test, were calculated according the trapezoidal rule.

HOMA-IR, homoeostasis model assessment of insulin resistance; HOMA-IR= FIns (mU/mL) ×FG (mmol/L)/22.5.

HOMA-β, homoeostasis model assessment of β-cell function; HOMA-β= FIns×20/(FG-3.5).

HDL, high-density lipoprotein; LDL, low-density lipoprotein; apo A-1, apolipoprotein A-1; apo B, apolipoprotein B; TG, Triglycerides.

*P_t-test_*, compared by independent Student’s *t*-test for the difference of baseline values between the two groups.

^a^ *p* < 0.05 by paired *t*-test with comparison of the difference between baseline and 12-week value.

^b^ *p* < 0.05 by independent Student’s *t*-test with comparison of the difference of net changes between the two groups.

**Table S5: Changes in adiponectin and glucolipid metabolic markers after 12-week intervention in** **subjects with prediabetes**

|  | **Anthocyanins (n=40)** | | **Placebo (n=36)** | | **Net change**  **(95% CI)** | ***P t-test*** |
| --- | --- | --- | --- | --- | --- | --- |
|  | **Baseline** | **12 weeks** | **Baseline** | **12 weeks** |  |  |
| Adiponectin (ug/mL) | 6.03±2.51 | 5.52±2.39 ^a^ | 6.53±2.12 | 6.37±2.25 | -0.35(-0.85, 0.16) | 0.174 |
| **Glucose metabolism** |  |  |  |  |  |  |
| Hemoglobin A1c (%) | 5.8±0.43 | 5.57±0.4 ^a^ | 5.68±0.45 | 5.54±0.5 ^a^ | -0.09(-0.2, 0.03) | 0.148 |
| Fasting glucose (mmol/L) | 5.68±0.69 | 6.01±0.62 ^a^ | 5.9±0.47 | 6.06±0.66 | 0.17(-0.19, 0.53) | 0.347 |
| 2-h glucose (mmol/L) | 7.57±2.02 | 9.28±2.55 ^a^ | 7.33±1.82 | 8.49±2.08 ^a^ | 0.56(-0.3, 1.41) | 0.197 |
| Fasting insulin (μU/mL) | 11.64±5.86 | 10.94±6.15 | 11.05±5.19 | 10.81±6.15 | -0.46(-2.13, 1.2) | 0.579 |
| 2-h insulin (μU/mL) | 81.74±54.9 | 105.38±79.91 ^a^ | 84.66±73.54 | 101.56±64.1 ^a^ | 6.74(-14.53, 28.02) | 0.529 |
| Fasting C-peptide (ng/mL) | 2.25±0.93 | 2.36±0.82 | 2.25±0.8 | 2.32±0.76 | 0.02(-0.24, 0.28) | 0.853 |
| 2-h C-peptide (ng/mL) | 10.87±3.72 | 11.57±4.39 | 10.69±3.71 | 11.78±3.95 ^a^ | -0.4(-1.68, 0.89) | 0.541 |
| AUC Glucose | 23.9±4.21 | 28.58±5.03 ^a^ | 24.18±3.74 | 26.72±4.32 ^a^ | 2.14(0.41, 3.86) ^b^ | 0.016 |
| AUC Insulin | 196.39±100.96 | 236.31±123.51 ^a^ | 205.41±106.26 | 256.85±149.88 ^a^ | -11.52(-46.37, 23.32) | 0.51 |
| AUC C-peptide | 25.28±7.66 | 26.52±7.79 | 25.44±6.24 | 27.83±7.98 ^a^ | -1.15(-3.44, 1.14) | 0.318 |
| HOMA-IR | 2.95±1.62 | 3±1.79 | 2.89±1.26 | 2.95±1.76 | -0.01(-0.59, 0.57) | 0.965 |
| HOMA-β | 113.43±69.66 | 90.01±45.87 ^a^ | 103.31±106.15 | 86.93±51.59 | -7.04(-38.31, 24.24) | 0.655 |
| **Lipids** |  |  |  |  |  |  |
| Total cholesterol (mmol/L) | 6.04±1.17 | 5.96±1.25 | 6.18±1.1 | 6.01±1.08 | 0.09(-0.37, 0.55) | 0.693 |
| Triglycerides (mmol/L) | 1.71±0.91 | 1.87±1.55 | 1.75±1.13 | 1.67±1.07 | 0.24(-0.24, 0.72) | 0.323 |
| HDL cholesterol (mmol/L) | 1.46±0.39 | 1.29±0.42 ^a^ | 1.5±0.36 | 1.31±0.31 ^a^ | 0.03(-0.11, 0.18) | 0.635 |
| LDL cholesterol (mmol/L) | 3.38±0.87 | 3.08±0.68 ^a^ | 3.36±0.91 | 3.26±0.71 | -0.2(-0.45, 0.05) | 0.119 |
| Apo A-1 (g/L) | 1.55±0.3 | 1.59±0.31 | 1.69±0.29 | 1.57±0.22 ^a^ | 0.15(0.03, 0.26) | 0.016 |
| Apo B (g/L) | 1.17±0.25 | 1.06±0.2 ^a^ | 1.16±0.24 | 1.15±0.23 | -0.1(-0.17, -0.03) | 0.008 |

Data are analyzed using the per-protocol data set and are presented as mean ±standard deviation.

AUC, area under the curve by 3-hour oral glucose tolerance test, were calculated according the trapezoidal rule.

HOMA-IR, homoeostasis model assessment of insulin resistance; HOMA-IR= FIns (mU/mL) ×FG (mmol/L)/22.5.

HOMA-β, homoeostasis model assessment of β-cell function; HOMA-β= FIns×20/(FG-3.5).

HDL, high-density lipoprotein; LDL, low-density lipoprotein; apo A-1, apolipoprotein A-1; apo B, apolipoprotein B; TG, Triglycerides.

*P_t-test_*, compared by independent Student’s *t*-test for the difference of baseline values between the two groups.

^a^ *p* < 0.05 by paired *t*-test with comparison of the difference between baseline and 12-week value.

^b^ *p* < 0.05 by independent Student’s *t*-test with comparison of the difference of net changes between the two groups.
